# 4‐Strand hamstring versus single‐bundle quadriceps tendon grafts in anterior cruciate ligament reconstruction at 2 years: A systematic review and meta‐analysis of randomised controlled trials

**DOI:** 10.1002/jeo2.70244

**Published:** 2025-04-22

**Authors:** Jonathan Elias, Mitchell Kaplan, Kunal Shah, Michael Bickford, Chelsea McNamara, Elizabeth Ford, Sean McMillan

**Affiliations:** ^1^ Rowan‐Virtua School of Osteopathic Medicine Stratford New Jersey USA; ^2^ Futures Forward Research Institute Toms River New Jersey USA; ^3^ Inspira Health Network Vineland New Jersey USA; ^4^ Virtua Health System Marlton New Jersey USA

**Keywords:** ACL, graft, hamstring, quadriceps, reconstruction

## Abstract

**Purpose:**

To compare the 2‐year postoperative outcomes of hamstring tendon (HT) grafts to quadriceps tendon (QT) grafts in anterior cruciate ligament (ACL) reconstruction. We hypothesised no significant differences between the two methods.

**Methods:**

A systematic review and meta‐analysis were conducted following the 2020 PRISMA guidelines. Five online databases (Cochrane, Embase, PubMed, Scopus and Web of Science) were screened for prospective randomised controlled trials (RCT). IKDC, Lysholm, Tegner, side‐to‐side KT‐1000 scores and ages were collected. Statistical analysis was conducted with SPSS v29. Between the two groups, a test of between‐subgroup homogeneity *p*‐value < 0.05 was used to assess statistical significance, while a *Δ* Cohen's *d* ≥ 0.8 was used to assess clinical significance.

**Results:**

Five RCTs were included in the final analysis. The HT group included 87 patients, and the QT group included 93 patients. The mean ages for the HT and QT groups were 28.3 ± 6.6 and 26.5 ± 8.4 years, respectively. HTs yielded clinically higher IKDC scores (*Δ* Cohen's *d* = 2.27, *p* = 0.24), clinically lower side‐to‐side KT‐1000 differences (*Δ* Cohen's *d* = 1.65, *p* = 0.08), and clinically and statistically higher Lysholm scores (*Δ* Cohen's *d* = 2.93, *p* = 0.00). However, QT yielded higher Tegner scores (*Δ* Cohen's *d* = 1.68, *p* = 0.00), whereas HT led to a moderate clinical reduction (Cohen's *d* = −0.55, *p* = 0.06, 95% CI [−1.11 to 0.02]).

**Conclusions:**

Compared with the use of the QT graft, the HT graft for ACL reconstruction may result in greater knee function and stability, while QT ACL reconstruction may be associated with increased levels of postsurgical activity in terms of return to sports and work. Future long‐term RCTs are needed to confirm our findings.

**Level of Evidence:**

Level I.

**Abbreviations**
ACLR: anterior cruciate ligament reconstruction
BPTB: bone patellar tendon bone
BTB: bone patella bone
HT: hamstrings tendon
IKDC: International Knee Documentation Committee
QT: quadriceps tendon

## INTRODUCTION

Injuries to the anterior cruciate ligament (ACL) affect 100,000–200,000 patients annually [14, 15]. In active patients, ACL reconstruction (ACLR) is indicated for increasing knee stability and improving clinical and functional outcomes [7]. The use of autografts versus allografts is dependent upon factors such as patient age, activity level, surgeon preference and revision cases [20]. Nevertheless, in younger patients, autografts have traditionally been indicated. In these patients, the bone patella bone (BTB), quadriceps tendon (QT) plus or minus bone plugs, and hamstring tendon (HT) represent the graft options for reconstruction.

Success rates across the literature for ACLR vary, with reported good to excellent results ranging from 75% to 97% [25]. While BTB has traditionally been the ‘gold standard’ of care, renewed interest in the use of QT and HT grafts has led to an increase in usage over the past decade. The benefits of using these grafts have been previously documented, including less postoperative pain and donor site morbidity [12].

In this systematic review and meta‐analysis, we compare 4‐stranded HT autografts to single‐bundle QT autografts. We focus on these two specific types of grafts as quadrupled HT grafts are among the most used in ACLR, while QT grafts have been gaining popularity as an alternative [1, 16]. Despite their widespread use, there remains ongoing controversy in the literature regarding which provides the most superior outcomes.

To our knowledge, the most recently conducted systematic review and meta‐analysis conducted on this topic was published in 2020; however, in the review there was a great range in when the outcomes were assessed postoperatively [21]. Additionally, we analysed a different set of studies, including more recently published trials [21]. The purpose of this review and meta‐analysis is to provide the most up‐to‐date comparison of 2‐year postoperative outcomes regarding 4‐strand HT autografts to single‐bundle QT autografts. It was hypothesised that there will be no difference in outcomes between HT ACLR and QT ACLR.

## METHODS

A systematic review and meta‐analysis were conducted following the 2020 Preferred Reporting Items for Systematic Reviews and Meta‐Analyses (PRISMA) guidelines [22]. The purpose of this study was to compare the 2‐year postoperative outcomes of 4‐strand HT grafts to single‐bundle QT grafts. Specifically, the following outcomes were compared: IKDC score, KT‐1000 side‐to‐side difference, Lysholm score, and Tegner score.

### Search procedure

A comprehensive review of five major scientific databases was performed to evaluate the outcomes of patients after ACLR with either an HT or QT autograft. On 16 June 2024, the PubMed, Embase, Scopus, Web of Science, and Cochrane databases were searched. The search string used across all databases was: (‘ACL Reconstruction’ OR ‘Anterior Cruciate Ligament Reconstruction’ OR ‘ACLR’) AND (‘Semitendinosus Tendon’ OR ‘Quadriceps Tendon’) AND (‘Graft’) AND (‘Randomized Controlled Trial’ OR ‘RCT’) with key terms identified via MeSH.

### Inclusion and exclusion criteria

The articles included in this meta‐analysis were only randomised controlled trials (RCT) with preoperative and postoperative measures assessed at 2 years postintervention. Articles that included ACL reconstruction using either a 4‐stranded semitendinosus tendon autograft (HT group) or single‐bundle quadriceps tendon autograft with or without a bone plug (QT group) were included. Studies excluded were any that were not an RCT, case studies, systematic reviews, and meta‐analyses. Articles that did not report the mean, standard deviation, or number of participants before and 2 years after the procedure were not included. Articles that did not use a 4‐stranded semitendinosus HT graft, a single‐bundle QT graft, or used allografts instead of autografts were excluded.

### Study selection

The studies were imported into Rayyan.ai, and independently screened by two authors (JE and MK), which led to the exclusion of 235 articles in total. Any disagreements between the original two authors were adjudicated by a third author (KS).

### Data extraction

Qualitative and quantitative data were collected after article selection was complete. The International Knee Documentation Committee (IKDC) score, KT‐1000 side‐to‐side difference, Lysholm score, and Tegner score were used as the primary outcomes of our study. If an RCT had multiple groups, only the data regarding 4‐stranded HT autografts and single‐bundle QT autografts were collected and included in our analysis.

### Statistical analysis

All data was collected independently by four reviewers (MK, KS, MB and CM). From each study, the baseline and 2‐year postoperative number of participants of each study were extracted, as well as the baseline and 2‐year postoperative means and standard deviations of the IKDC scores, KT‐1000 side‐to‐side differences, Tegner scores, and Lysholm. Statistical analysis was performed via a meta‐analysis with a random effects model using IBM SPSS Statistics for Windows, version 29 (IBM Corp., Armonk, N.Y., USA). The meta‐analysis pooled the effect sizes of all the articles, allowing us to evaluate the mean changes in the postoperative outcomes compared with the preoperative assessments. The extent of improvement in the postoperative outcomes was determined by the effect size of the analysis (Cohen's *d*) with 95% confidence intervals. A test of between‐subgroup homogeneity with a *p*‐value < 0.05 was used to determine the statistical significance between the outcomes of the two groups [24]. A *Δ* Cohen's *d* between the two groups ≥ 0.8 was used to determine clinical significance [28].

Heterogeneity, or the variances between the studies chosen the *I* ^2^ ratio (*I* ^2^ = *τ* ^2^/*H* ^2^). A larger *I* ^2^ value suggests variance between the studies, which could be due to bias or another variable. Tau‐squared (*τ* ^2^) represents the variation between effect sizes, without considering the variation expected from random chance. A random effect model was used, allowing for analysis of variance that was expected to be greater than chance to determine the effect of an external variable compared with the dependent variable. *H* ^2^ was determined to analyse variance, with a value of 1 indicating an equivalent variance between the fixed and random effect models, which would represent low heterogeneity. Low heterogeneity implies that there is a minor difference between the changes in the study's effect size compared with if there was only random chance.

### Risk of bias and certainty of evidence assessment

The methodological quality of the articles included in this meta‐analysis was evaluated via the modified Grading of Recommendations Assessment, Development and Evaluation (GRADE) criteria (Table 2). Bias in the articles was assessed by two authors (JE and KS). Because all the articles included were RCTs, the articles were subjected to evaluation according to RoB‐2.

## RESULTS

### Search results

**Figure 1:**
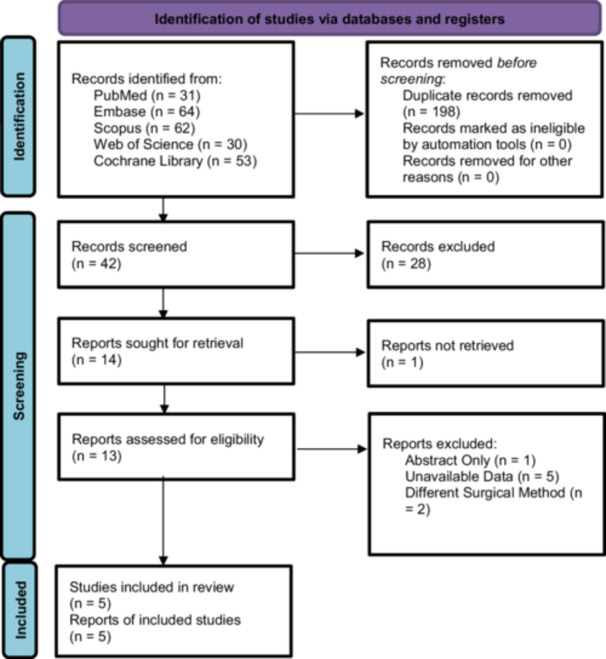
PRISMA flowchart of article selection [10].

A total of 240 articles were imported into Rayyan.ai for the detection and deletion of duplicates. The detected duplicates were confirmed before deletion. A total of 198 duplicates were removed, leaving 42 article titles and abstracts to be screened using the inclusion and exclusion criteria. Thirteen articles were included in the full article review, with articles being excluded for not having the data available (*n* = 5), performing a surgery that the authors were not looking at (*n* = 2), or were abstracts only (*n* = 1). A flow chart of the selection process has been presented in Figure 1. Two prospective studies that may seem to fit the criteria but were excluded because they were nonrandomized were the studies conducted by Bastidas et al. and Cavaignac et al. [4, 23]. After thorough examination of the literature, five randomised trials were included in the final analysis.

### Group demographics

The HT group included 87 patients, and the QT group included 93 patients, yielding 180 distinct patient evaluations assessed at 2‐years postoperation. The mean ages for the HT and QT groups were 28.3 ± 6.6 and 26.5 ± 8.4 years, respectively.

### Summary of findings

Three of the five studies focused on the use of the QT for ACLR, two of which utilised bone, whereas Ebert et al. [5] used only soft tissue. Irrgang et al. [9] conducted an RCT comparing the use of single‐bundle (*n* = 28) versus double‐bundle (*n* = 29) quadriceps tendon grafts, using a bone plug in all cases. The study assessed outcomes such as IKDC, KOOS, KT‐100, and pivot tests measured at 2 years postoperatively. No measurable differences were found between the two groups due to early termination. Lund et al. [13] performed a comparative analysis of bone‐patellar tendon‐bone (BPTB) (*n* = 25) and quadriceps tendon patellar‐bone (*n* = 26), with bone plugs being used in all cases. The outcomes of KT‐1000, KOOS, and IKDC scores were compared, portraying no significant differences between the two. Ebert et al. [5] compared the use of HT (*n* = 55) and QT (*n* = 57) autografts with outcomes measured at 3‐, 6‐, 12‐, and 24‐month intervals. Except for returning to sports after injury, which was significantly different among the HT group, all other measured outcomes, such as the IKDC score, Lysholm score, Cincinnati score, Knee Outcome Survey score, and Tegner score, were not significantly different.

Two studies fit the eligibility criteria for HT autografts. Bi et al. [2] compared the outcomes of the anterior half of the peroneus longus tendon (AHPLT; *n* = 62) and the semitendinosus (*n* = 62) with the assessments of IKDC score, KT‐1000, pivot test, and Visual Analogue Scale. The semitendinosus group utilised an all‐inside technique. The data analysed revealed no significant differences between the two groups, suggesting that the AHPLT is a viable alternative to semitendinosus for ACLR. Streich et al. [27] provided a comparative analysis for 4‐strand single‐bundle reconstruction with an HT graft (*n* = 25) using the complete tibial tunnel technique, versus a 2‐strand HT graft with a double bundle (*n* = 24). For the outcomes of the IKDC score, Lysholm score, Tegner activity score, KT‐1000, and pivot‐shift test, each technique yielded significant results compared with the baseline; however, no significant results were obtained between the two techniques.

### Complication rates (Table 1)

#### IKDC score

**Figure 2:**
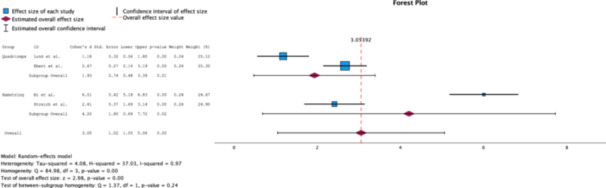
Forest plot comparing the effect size of QT grafts to HT grafts on IKDC scores measured at 2‐ years postoperatively [2, 5, 9, 13, 27]. HT, hamstrings tendon; IKDC, International Knee Documentation Committee; QT, quadriceps tendon.

A random effects analysis of the IKDC scores of each group revealed clinical significance (*Δ* Cohen's *d* = 2.27) in favour for the HT group, with Cohen's *d* = 4.20 (95% CI [0.69–7.72]), while the QT group portrayed a Cohen's *d* = 1.93 (95% CI [0.48–3.39]). However, no statistically significant difference (*p* = 0.24) was found between the groups (Figure 2).

**Table 1:** Summary of complication rates from each included study.

Group	Author	Complication rates	Failure rates
Quadriceps grafts	Irrgang et al. [8] (*N* = 28)	Meniscal tear: 4 (14.3%) Patellar fracture: 3 (10.7%) Unspecified knee pain: 3 (10.7%) Suture abscess: 4 (14.3%) Surgical fixation for patellar fracture: 1 (3.6%) Quadriceps weakness: 1 (3.6%) Contralateral ACL tear: 2 (7.1%) Fall: 1 (3.6%) Abnormal rotatory laxity: 2 (7.1%) Deep vein thrombosis: 1 (3.6%)	Ipsilateral ACL re‐tear: 1 (3.6%) MRI evidence of graft failure: 1 (3.6%)
	Lund et al. [13] (*N* = 25)	Superficial infection: 1 (4%) Screw Protrusion: 1 (4%) Inflamed medial plica: 1 (4%)	No graft failure
	Ebert et al. [5] (*N* = 57)	ROM impact: 1 (1.8%) Scar tissue: 1 (1.8%) Meniscal repair: 2 (3.5%) Tibial tubercle transfer: 1 (1.8%) Cartilage injury: 1 (1.8%) Contralateral ACL tear: 2 (3.5%)	Ipsilateral ACL re‐tear: 1 (1.8%)
Hamstrings grafts	Bi et al. [2] (*N* = 62)	N/A	N/A
	Streich et al. [27] (*N* = 25)	Mild pain at graft site: 3 (12%)	No graft failure
	Ebert et al. [5] (*N* = 55)	ROM impact: 2 (3.6%) Scar tissue: 2 (3.6) Meniscal repair: 1 (1.8%)	No graft failure

Abbreviations: ACL, anterior cruciate ligament; MRI, magnetic resonance imaging; ROM, range of motion.

#### KT‐1000 side‐to‐side difference

**Figure 3:**
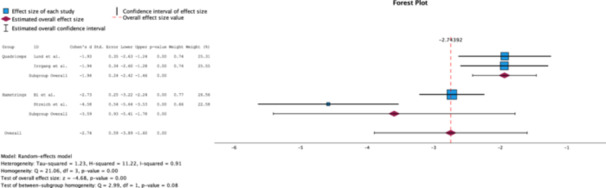
Forest plot comparing the effect size of QT grafts to HT grafts on KT‐1000 side‐to‐side scores measured 2 years postoperatively [2, 5, 9, 13, 27]. HT, hamstrings tendon; QT, quadriceps tendon.

A random effects analysis of the KT‐1000 side‐to‐side differences among the groups revealed that the HTs (Cohen's *d* = −3.59, 95% CI [−5.41, −1.78]) were clinically superior (*Δ* Cohen's *d* = 3.47) than the QTs were (Cohen's *d* = −1.94, 95% CI [−2.42, −1.46]). However, the results did not portray statistical significance (*p* = 0.08) (Figure 3).

#### Lysholm score

**Figure 4:**
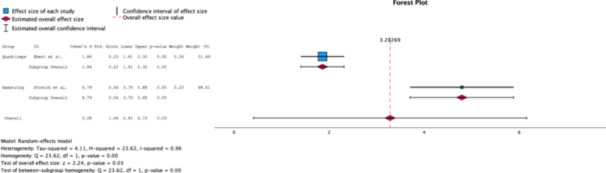
Forest plot comparing the effect size of QT grafts to HT grafts on Lysholm scores measured at 2 years postoperatively [2, 5, 9, 13, 27]. HT, hamstrings tendon; QT, quadriceps tendon.

A random effects analysis revealed that the HT group (Cohen's *d* = 4.79, 95% CI [3.70–5.88]), yielded both clinically (*Δ* Cohen's *d* = 2.93) and statistically significant (*p* = 0.00) increases in the Lysholm score, compared to the QT group (Cohen's *d* = 1.86, 95% CI [1.41–2.32]) (Figure 4).

#### Tegner activity score

**Figure 5:**
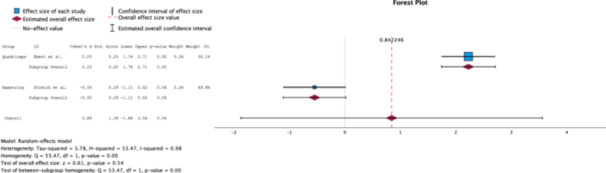
Forest plot comparing the effect size of QT grafts to HT grafts on Tegner scores measured at 2 years postoperatively [2, 5, 9, 13, 27]. HT, hamstrings tendon; QT, quadriceps tendon.

A random effects analysis of the Tegner score revealed a clinical (*Δ* Cohen's *d* = 1.68) and statistical significance (*p* = 0.00) in the QT group (Cohen's *d* = 2.23, 95% CI [1.74–2.71] than the HT group (Cohen's *d* = −0.55, 95% CI [−1.11, 0.02]) (Figure 5).

### Risk of bias and certainty of evidence assessment

The grade analysis for the certainty of the evidence was performed by two independent authors (JE and KS) and yielded four moderate‐level studies and one low‐level study. Primarily, indirectness was affected since none of the studies provided a direct comparison between the two subgroups. One study was stopped in the middle of recruitment, providing imprecise results, whereas other studies had suspected publication bias. An overall grade analysis is presented in Table 2.

**Table 2:** Overall GRADE analysis of the included studies.

Modified grading of recommendations assessment, development, and evaluation criteria for included articles								
Author	Study design	Risk of bias	Inconsistency	Indirectness	Imprecision	Publication bias	Other factors	Final grade
Irrgang et al. [9]	RCT	Serious	Not serious	Serious	Very serious	Undetected	N/A	Low
Bi et al. [2]	RCT	Not serious	Serious	Serious	Not serious	Undetected	N/A	Moderate
Lund et al. [13]	RCT	Not serious	Serious	Serious	Serious	Undetected	N/A	Moderate
Streich et al. [27]	RCT	Not serious	Not serious	Serious	Not serious	Strongly suspected	N/A	Moderate
Ebert et al. [5]	RCT	Serious	Not serious	Not serious	Not serious	Strongly suspected	N/A	Moderate

Abbreviations: GRADE, Grading of Recommendations Assessment, Development and Evaluation; RCT, randomised controlled trials.

**Figure 6:**
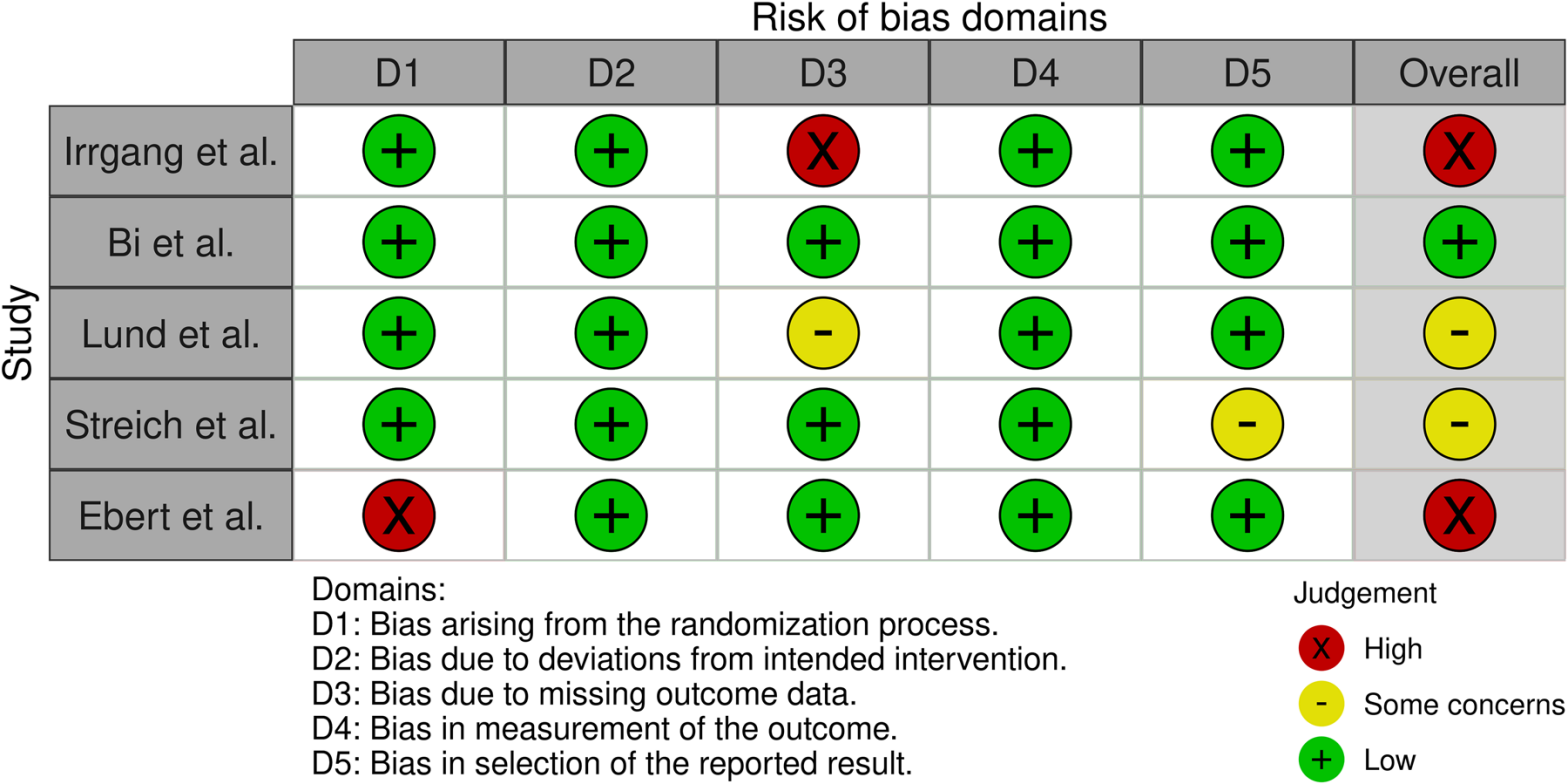
Traffic light plot portraying the RoB‐2 assessment.

**Figure 7:**
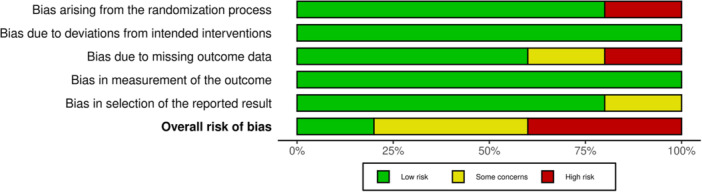
RoB‐2 summary plot.

A risk of bias assessment for each of the included studies was performed based on the Cochrane Risk of Bias Handbook and tool by the same two authors [26]. A RoB‐2 analysis was performed since the studies included were all RCTs, with two studies being high risk, two with some concerns and one study with a low risk of bias. Each domain of RoB‐2 is represented in Figures 6 and 7 via the *ROBVIS* tool [17].

## DISCUSSION

This systematic review and meta‐analysis serves as the most up‐to‐date collection of data comparing the two‐year outcomes of using either a 4‐strand HT graft or a single‐bundle QT graft in ACL reconstruction.

Our results show that the use of the HT graft yields clinically higher IKDC scores, clinically lower side‐to‐side KT‐1000 differences, and clinically and statistically higher Lysholm scores. On the other hand, using a QT graft resulted in clinically and statistically higher Tegner scores, whereas the HT graft led to a moderate clinical decrease compared with baseline. These findings nullify our original hypothesis.

Although subjective, the IKDC score is used as a reliable, consistent, and valid method to accurately quantify outcomes following ACL reconstruction [6, 8, 10]. The IKDC assesses overall knee functionality by accounting for patient‐reported activity in sports, function, and measures of symptoms [6]. Similarly, the Lysholm knee scoring scale assesses patients’ degree of restraint, limping, oedema, support, instability, pain, ability to ascend through stairs, and squatting [3]. Utilisation of a semitendinosus tendon graft may have yielded higher IKDC and Lysholm scores than did the quadriceps graft for a variety of reasons. One possibility may be simple biomechanics, as the primary extensors of the knee are the quadriceps, patella, and patellar tendon: all of which may have been compromised during the procedure, especially since two of the included quadriceps studies utilised a bone plug. On the other hand, the knee has multiple flexors, such as the biceps femoris, semimembranosus, gracilis, gastrocnemius, and popliteus, all of which can act as collateral if the semitendinosus is weakened. Additionally, with two of the studies utilising a QT graft with a bone plug, the relatively diminished effect on IKDC and Lysholm scores in the QT graft may be partly due to the postoperative weakened structural integrity of the patella, as a systematic review conducted by Meena et al. [19] concluded that the harvesting of a patellar bone block in ACL reconstruction is correlated with a greater risk of patellar fracture.

To determine the laxity of the ACL, side‐to‐side assessments via a KT‐1000 arthrometer were performed before and 2 years after surgery. The use of the HT graft had a stronger effect size on reducing the differences in side‐to‐side laxity, resulting in laxity closer to the physiological response of the unoperated knee.

The Tegner activity score assesses patients' activity in work and sports and is often utilised in conjunction with the Lysholm scale, as the Lysholm scale may mask the inactivity of patients [18]. In our analysis, we observed that the use of the QT graft in ACL reconstruction resulted in higher Tegner scores than the HT group, with the HT group reporting a moderate clinical decrease in activity 2 years postoperatively compared with preoperative levels. It is possible that the Lysholm scores masked the inactivity of the patients, as previously described, resulting in this discrepancy between the Lysholm and Tegner scores. Given that Tegner scores are a predictive measure of return to sports, athletes may be in the best interest of utilising a QT graft during ACL reconstruction [11]. However, given the comparison of only two studies for the Tegner score, a definite conclusion cannot be drawn.

When comparing the HT and QT ACLR techniques, greater knee functionality, and improved stability postoperation were reported among those who underwent an HT graft. In contrast, patients who underwent a QT graft reported higher levels of activity in work and sports than did the HT graft patients. However, owing to the high degree of heterogeneity, the physician must use their own discretion to select the best ACLR procedure. The findings presented in this review warrant further long‐term head‐to‐head randomised trials to establish which graft is superior. Physicians should continue studying the outcomes of each ACLR technique to determine the long‐term effects of each treatment. These include the use of knee biomechanics, KT‐1000 scoring, and self‐report questionnaires such as the Tegner, Lysholm, and IKDC scores. This review and meta‐analysis may help guide orthopaedic surgeons in choosing the best ACLR technique for individual patients.

A recent systematic review and meta‐analysis that included RCTs, prospective studies, and retrospective studies, analysed that the use of QT was associated with having a reduced rate of graft site failure and a lower KT‐1000 side‐to‐side difference in relation to HT grafts, while portraying a reduced risk of donor site pain and moderate‐to‐severe kneecap symptoms compared to BPTB grafts [29]. Our results regarding the KT‐1000 differences seem to be contradictory, possibly due to our review only including RCTs.

### Limitations

There are several limitations to this review. The values generated from the IKDC meta‐analysis, *τ* ^2^ = 4.08, *H* ^2^ = 37.03, and *I* ^2^ = 0.97, revealed significant heterogeneity among the included studies. As there was a clinically significant difference in outcomes between the HT and QT groups in the IKDC analysis, the results are expected to portray high heterogeneity. One possible source of heterogeneity intrinsic to the set‐up of our review may be that two of the three studies utilizing the QT graft included a bone plug in all the cases, whereas the paper written by Ebert et al. [5] strictly used an all soft‐tissue approach. However, a recently published systematic review and meta‐analysis states that both methods yield comparable outcomes, revision rates, and complications, so it is unlikely that including both bone plugs and all soft‐tissue grafts within the QT graft contributed strongly to variance [19]. Regarding the HT group, the study conducted by Bi et al. [2] used an all‐inside approach, while the Streich et al. [27] study utilised the complete tibial tunnel method, which may have added to the variability of the review. Additionally, other factors, such as time from injury to procedure, mean age, and surgeon experience between studies added to the heterogeneity within the review. Furthermore, two out of five of the utilised studies portrayed a high risk of bias according to the RoB‐2 assessment. Lastly, complication and reoperation rates were unable to be compared between the two groups, due to inadequate data in the included RCTs. Given the high amount of variability between the included studies, the generalisability of our review is limited.

## CONCLUSION

Compared with the QT graft, the findings of this study suggest that the use of the HT graft for ACL reconstruction may be associated with greater knee function and stability, while use of the QT may be associated with increased levels of postsurgical activity in terms of return to sports and work. While each type of graft showed their own respective favourable short‐term outcomes, further long‐term studies are needed to confirm these findings and assess functional outcomes beyond two years.

## AUTHOR CONTRIBUTIONS

Jonathan Elias conceived of the presented idea. Jonathan Elias, Mitchell Kaplan, and Kunal Shah selected studies. Mitchell Kaplan, Kunal Shah, Michael Bickford, and Chelsea McNamara extracted data from the studies included. Jonathan Elias completed the statistics. Jonathan Elias and Kunal Shah completed the GRADE and bias assessment for each included study. Jonathan Elias, Mitchell Kaplan, Kunal Shah, Michael Bickford, Chelsea McNamara, Elizabeth Ford, and Sean McMillan participated in the write‐up of the manuscript. Elizabeth Ford and Sean McMillan reviewed the study concept, provided areas for improvement of the study concept, and reviewed the manuscript.

## CONFLICT OF INTEREST STATEMENT

Dr. McMillan reports a relationship with Trice Medical that includes: consulting or advisory and speaking and lecture fees. Dr. McMillan reports a relationship with Arthrex Inc that includes consulting or advisory. All other parties do not have any potential conflicts of interest to disclose.

## ETHICS STATEMENT

This systematic review and meta‐analysis does not require an IRB approval/exemption as there was no interaction with human subjects or access to private information. The review was not registered with an institutional review board, as there was no contact with human subjects or access to personal information. A protocol was prepared for the review but has not been published. The protocol was followed as planned with no amendments.

## Supporting information

Supporting information.

## Data Availability

The data extracted from each study is publicly available at each study's respective journal.
